# Web-Based TangPlan and WeChat Combination to Support Self-management for Patients With Type 2 Diabetes: Randomized Controlled Trial

**DOI:** 10.2196/30571

**Published:** 2022-03-30

**Authors:** Shu-Fang Xia, Gusonghan Maitiniyazi, Yue Chen, Xiao-Ya Wu, Yu Zhang, Xiao-Yan Zhang, Zi-Yuan Li, Yuan Liu, Yu-Yu Qiu, Jun Wang

**Affiliations:** 1 Wuxi School of Medicine, Jiangnan University Wuxi China; 2 Department of Rehabilitation, Wuxi 9th Affiliated Hospital of Soochow University Wuxi China; 3 Department of Endocrinology, Affiliated Hospital of Jiangnan University Wuxi China; 4 Department of Hepatobiliary Surgery, Affiliated Hospital of Jiangnan University Wuxi China

**Keywords:** type 2 diabetes, glucose control, TangPlan, WeChat, self-management

## Abstract

**Background:**

China has the largest number of patients with type 2 diabetes mellitus (T2DM) in the world. However, owing to insufficient knowledge of self-management in patients with diabetes, blood glucose (BG) control is poor. Most diabetes-related self-management applications fail to bring significant benefits to patients with T2DM because of the low use rate and difficult operation.

**Objective:**

This study aims to examine the effectiveness of the combination of the self-designed web-based T2DM management software TangPlan and WeChat on fasting BG (FBG), glycated hemoglobin (HbA_1c_), body weight, blood pressure (BP), and lipid profiles in patients with T2DM over a 6-month period.

**Methods:**

Participants were recruited and randomized into the TangPlan and WeChat or control groups. Participants in the control group received usual care, whereas the TangPlan and WeChat participants received self-management guidance with the help of TangPlan and WeChat from health care professionals, including BG self-monitoring; healthy eating; active physical exercise; increasing medication compliance; and health education during follow-ups, lectures, or web-based communication. They were also asked to record and send self-management data to the health care professionals via WeChat to obtain timely and effective guidance on diabetes self-management.

**Results:**

In this study, 76.9% (120/156) of participants completed the 6-month follow-up visit. After the intervention, FBG (mean 6.51, SD 1.66 mmol/L; *P*=.048), HbA_1c_ (mean 6.87%, SD 1.11%; *P*<.001), body weight (mean 66.50, SD 9.51 kg; *P*=.006), systolic BP (mean 127.03, SD 8.00 mm Hg; *P*=.005), diastolic BP (mean 75.25, SD 5.88 mm Hg; *P*=.03), serum low-density lipoprotein cholesterol (mean 2.50, SD 0.61 mmol/L; *P*=.006), and total cholesterol (mean 4.01, SD 0.83 mmol/L; *P*=.02) in the TangPlan and WeChat group were all significantly lower, whereas serum high-density lipoprotein cholesterol (mean 1.20, SD 0.25 mmol/L; *P*=.01) was remarkably higher than in those in the control group. Compared with the baseline data, significance was found in the mean change in FBG (95% CI −0.83 to −0.20; *P*=.002), HbA_1c_ (95% CI −1.92 to −1.28; *P*<.001), body weight (95% CI −3.13 to −1.68; *P*<.001), BMI (95% CI −1.10 to −0.60; *P*<.001), systolic BP (95% CI −7.37 to −3.94; *P*<.001), diastolic BP (95% CI −4.52 to −2.33; *P*<.001), triglycerides (95% CI −0.16 to −0.03; *P*=.004), serum low-density lipoprotein cholesterol (95% CI −0.54 to −0.30; *P*<.001), and total cholesterol (95% CI −0.60 to −0.34; *P*<.001) in the TangPlan and WeChat group but not in the control group (*P*=.08-.88).

**Conclusions:**

Compared with usual care for patients with T2DM, the combination of TangPlan and WeChat was effective in improving glycemic control (decrease in HbA_1c_ and BG levels) and serum lipid profiles as well as reducing body weight in patients with T2DM after 6 months.

**Trial Registration:**

Chinese Clinical Trial Registry ChiCTR2000028843; https://tinyurl.com/559kuve6

## Introduction

### Background

In recent years, diabetes mellitus (DM) has gradually become an epidemic worldwide. China is the epicenter of the diabetes epidemic worldwide with an estimated 114.4 million people with diabetes in 2017 [1], mainly type 2 DM (T2DM). In a nationally representative cross-sectional survey conducted in 2013 in mainland China, the estimated overall prevalence of diabetes and prediabetes was 10.9% and 35.7%, respectively [2]. It has been suggested that medical management of diabetes alone before complications already accounts for 8.5% of national health expenditure in China, which puts heavy financial burdens on the country and patients [3]. Despite the development of therapeutic drugs and advanced techniques, the blood glucose (BG) levels of patients with DM are still poorly controlled [4], leading to various complications [5]. Self-management is considered the most critical factor in ensuring well-controlled BG levels, thereby preventing DM complications [6,7]. Self-management includes tracking BG trends, adhering to medication, monitoring nutrition, and increasing physical activity based on good diabetes health education [8]. Continuous DM care needs effective self-management education and support for both patients and family members.

Previous studies have found that increasing communication with health care professionals and enhancing diabetes management are beneficial for glycemic control [9]. However, Chinese physicians are usually very busy at work; patients only have a few minutes to consult physicians and fail to gain detailed DM self-management knowledge in a limited time. Furthermore, acute diabetic conditions and related chronic conditions force physicians to solve at least two symptomatic problems during the visit rather than the more time-consuming management of diabetes [10]. Only 20% of physicians consider they have the necessary resources to effectively manage patients with diabetes [11] and, during a visit, diabetes-specific assessments such as BG monitoring trends, history of hypoglycemia, foot examination, and blood pressure (BP) are not always performed. In addition, few patients have diabetes diaries, which prevents physicians from providing effective treatment guidance [12].

Growing evidence suggests that emerging telemedicine may further improve diabetes self-management and clinical outcomes via the establishment of an active interaction between patients and health care professionals. Apps are feasible tools to improve self-management of T2DM [13,14], resulting in positive self-management behaviors such as appropriate diet, enhanced physical activity, and BG monitoring [15]. However, despite these positive outcomes, the reality is that only a small proportion of patients use apps for diabetes self-management in China. In particular, older adults with diabetes, who account for 80.8% of the total number of patients with diabetes [16], are unable to use all kinds of professional apps well, resulting in poor use of apps for diabetes self-management. Moreover, most of the apps are completely new, which is not easy for middle-aged and older Chinese adults to use. Even in high-income countries, diabetes app use rate is still low. In Australia, only 8% of patients with diabetes use apps to support diabetes self-management [17]. Furthermore, owing to the lack of interconnected internetworking systems in different hospitals across China, it is very difficult for health care professionals to obtain the patient’s diagnosis and treatment records from other hospitals and continuous follow-up information on patients.

With the development of mobile health, the ways of acquiring medical consultations have changed. WeChat is an extremely popular social app in China, and it is also simple for older adults to operate. Many researchers have reported the effectiveness of WeChat in chronic disease management, including diabetes, hypertension, cancer, obesity, stable coronary artery disease, and chronic obstructive pulmonary disease [18-21]. However, it is also challenging for health care professionals to give patients with diabetes detailed self-management advice based only on WeChat, including diet and exercise advice. Therefore, we designed a diabetes management software for health care professionals, TangPlan, which is based on Chinese culture and can be used in conjunction with WeChat to provide detailed diabetes self-management advice.

### Objective

The primary objective of this study is to estimate the impact of the combination of TangPlan and WeChat on BG, glycated hemoglobin (HbA_1c_), body weight, BP, and lipid profiles in patients with T2DM for a 6-month period.

## Methods

### Study Design

This study was designed as a 6-month, nonblinded randomized controlled trial (ChiCTR2000028843) between April 1, 2020, and October 31, 2020, to examine the efficacy and feasibility of the combination of the web-based TangPlan and WeChat on BG control in patients with T2DM. Potential participants registered in the community who received the notification calls about the trial and came for clinic visits were identified by trained health workers in a community health care center in Wuxi, China. These participants had established a health record in the community health care center before January 2020. The CONSORT-EHEALTH (Consolidated Standards of Reporting Trials of Electronic and Mobile Health Applications and Online Telehealth) checklist is presented in Multimedia Appendix 1.

### Ethics Approval

This study was approved by the Jiangnan University Medical Ethics Committee (JNU20200312IRB04). The study was conducted in accordance with the CONSORT (Consolidated Standards of Reporting Trials) ethical guidelines and the CONSORT-EHEALTH checklist [22]. All researchers involved had been trained uniformly before the trial started to ensure that they were familiar with the trial procedures and methods.

### Participants

All participants confirmed their willingness to participate in face-to-face screening interviews to assess their eligibility. Our health care professionals recorded the participants’ relevant information, such as the type of disease, cognitive function, literacy capacity, surgery history in the past 6 months, planned residence time in the city, and mobile phone operation ability. After that, based on the inclusion and exclusion criteria, our health care professionals selected eligible participants for enrollment.

The criteria for inclusion in the study were as follows: (1) patients diagnosed with T2DM who met the 1999 World Health Organization diagnostic criteria; (2) participants with normal cognitive function who could read and write and voluntarily participated in the study; (3) no history of major surgery during the past 6 months, no major surgery plan in the next 6 months, and absence of any medication condition that could prevent the patients from walking for 15 to 30 minutes a day; (4) participants who had lived in Wuxi for more than half a year and were willing to participate in regular follow-ups; and (5) participants or family members living with them who could use WeChat proficiently, including sending and receiving messages, voice calls, and video calls.

The exclusion criteria for enrollment were as follows: (1) diagnosis of type 1 DM, gestational DM, maturity-onset diabetes of the young, or any other type of diabetes; (2) patients undergoing hemodialysis for chronic kidney disease; (3) history of any serious heart-related events (such as heart attack or stroke) in the past year; (4) pregnant patients or patients planning for a pregnancy in the next 6 months; (5) patients with disturbance of consciousness and mental disorders; and (6) patients participating in other intervention studies.

The participants received no compensation but were enrolled in the program for free. Before participating in the program, informed consent was obtained from each participant to use their data for clinical research.

### Sample Size

The sample size was calculated based on a completely random design using the sample size formula for the comparison of the mean of 2 independent samples. The trial was designed for analysis using 2-tailed tests, with type I and II error rates set at 0.05 and 0.1, respectively. We used the difference in the mean HbA_1c_ (0.91%) between the intervention and control groups along with the SD (1.14 for the intervention group and 1.61 for the control group) from a study on diabetes education and SMS text messaging reminders on metabolic control and disease management in patients with T2DM [23]. The latter study was similar to our trial as both were randomized controlled trials with primary outcomes of HbA_1c_. The calculations indicated that the total sample size required for each group was 50. Considering a dropout rate of up to 20%, the final sample size was determined to be ≥60 cases in each group.

### Program

#### Diabetes Self-management Education Team

Our multidisciplinary team comprised health care professionals, including the general physician from the community health care center, a diabetes specialist nurse, physicians from the department of endocrinology, physicians from the department of rehabilitation, a dietitian, and trained diabetes health educators.

#### TangPlan Software

**Figure 1:**
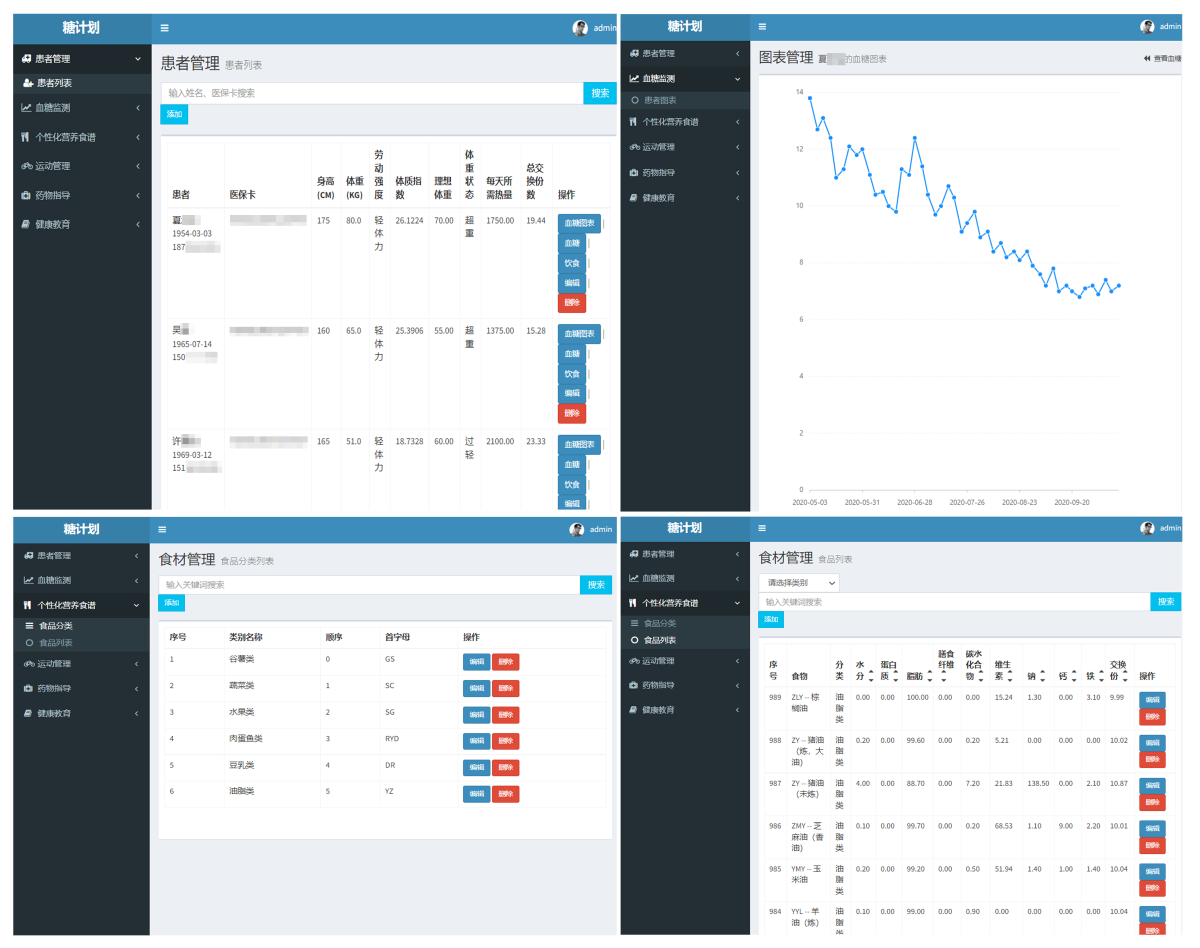
TangPlan website.

The diabetes management software TangPlan (Figure 1) was designed by our multidisciplinary team using the focus group method, and technical support was provided by Wuxi Wutong Leaf Technology Co, Ltd. TangPlan includes 6 functional modules (Textbox 1).

**The 6 functional modules included in TangPlan.**

1. Patient list module: Each patient’s information includes name, sex, date of birth, health insurance card number, mobile phone number, education level, food allergy, religious beliefs, normal exercise type and duration, body height, body weight, BMI, physical activity (intensity grades include very light, light, middle, and heavy), type 2 diabetes mellitus (T2DM) duration in years, glycated hemoglobin (HbA_1c_), systolic blood pressure, diastolic blood pressure, fasting blood glucose (BG), 2-hour postload BG, serum triglycerides, high-density lipoprotein cholesterol, low-density lipoprotein cholesterol, total cholesterol, medication (oral hypoglycemic agents, insulin, or both), and comorbidity (including hypertension, hyperlipidemia, coronary heart disease, cerebral infarction, and myocardial infarction).
2. BG monitoring module: The patient’s BG level is recorded in this module, and a dynamic BG chart is generated.
3. Personalized nutrition customization module: In the food management submodule, all ingredients are divided into six categories: cereals, vegetables, fruits, beans and bean products, meat and fish, and oil. Each ingredient has an exact nutritive value, including water; protein; fat; dietary fiber; carbohydrates; and vitamins, calories, sodium, calcium, iron, glycemic index, and glycemic load. In the recipe design submodule, based on the patient’s personalized information from the patient list module and BG monitoring module, the calorie requirement and the dietary recipe are automatically designed for each patient by a general physician, a physician from the department of endocrinology, or a dietitian. The software can automatically generate a weekly recipe and does not use ingredients with a higher glycemic index and load, and some ingredients can be adjusted based on the patient’s willingness.
4. Physical activity module: Physical activity includes aerobic and resistance exercises. Aerobic exercises include walking, jogging, long-distance swimming, cycling, tai chi, and fitness dance. Resistance exercises include push-ups, sit-ups, squats, barbell curls, upright lifts, bend lifts, bench presses, and overhead presses. According to the participant’s previous exercise experience, physicians from the department of endocrinology and rehabilitation select the appropriate physical activity and ascertain the exercise time after careful assessment.
5. Medication guidance module: According to the patient’s economic and medical conditions combined with the patient’s medication history, the patient’s medication situation can be recorded. In addition, a physician from the department of endocrinology provides reasonable medication guidance.
6. Health education module: The T2DM-related knowledge misunderstandings of each patient are recorded so that our team members, especially the diabetes specialist nurse and trained diabetes health educators, can correct them during diabetes health education.

### Allocation

After the participants were finally determined to be eligible to participate in this research, they were notified to go to the community health care center at a designated time and digitally randomized in a 1:1 allocation ratio either to the combination of TangPlan and WeChat group or usual care alone (control) group. After the results of the grouping were released, the participants were not allowed to switch groups.

### Intervention and Control Groups

#### Diabetes Usual Care (Control Group)

Participants in the control group went to the diabetes clinic of the community health care center and received usual care, including medication adjustment, guidance on a healthy and reasonable diet, suggestions on BG self-monitoring, and physical activity.

#### Combination of TangPlan and WeChat Care (TangPlan and WeChat Group)

**Figure 2:**
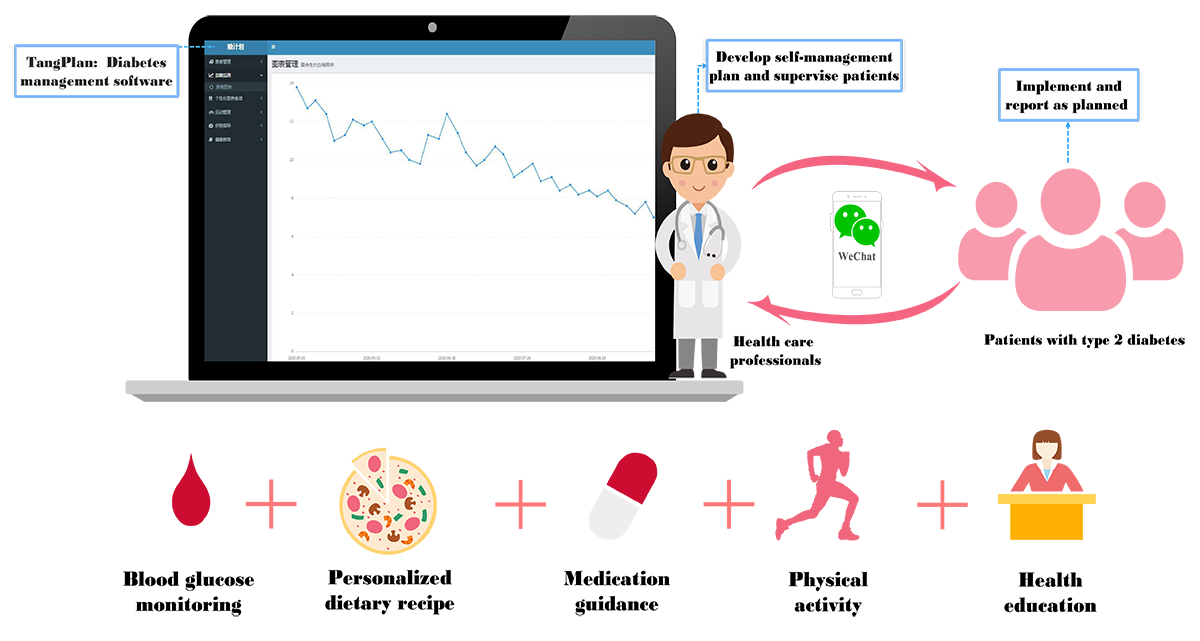
The general framework of the program.

Our team members collected all the information needed of each TangPlan and WeChat group participant in the TangPlan patient list module before the program started. During the first visit of the TangPlan and WeChat participants, we added them as WeChat friends, created a WeChat group, and ensured that the participants could use it proficiently. The program coached the participants in five areas: improving BG self-monitoring, healthy eating, active physical exercise, increasing medication compliance, and health education (Figure 2).

In terms of BG self-monitoring, we asked the participants about the frequency of BG monitoring at home and evaluated the effects of medication, diet, and physical activity on BG control. We then gave recommendations on the BG monitoring time and frequencies of the participants with T2DM. We also told the participants that, once they tested their BG at home, they should send the data to us via WeChat, and then we could record it in TangPlan and provide personalized recommendations.

Next, we used TangPlan to automatically design a personalized weekly diet plan, adjusted some ingredients based on the participant’s willingness, and finally printed the dietary recipe for the participant (Multimedia Appendix 2). We formulated the various ingredients that the participants needed every day, and the combination of ingredients and cooking methods was determined by the participants themselves. The participants generally thought that they could accept the dietary recipes. We encouraged the participants to keep a diet diary and bring it during follow-ups.

Physical activity was carried out under the guidance of physicians from the departments of endocrinology and rehabilitation. They performed medical assessments of cardiopulmonary and exercise function before physical activity. For general patients with T2DM, the goal was to exercise ≥5 days a week with 30 minutes of aerobic exercise each time, including tai chi and walking. If the participant had no contraindications, resistance exercise should be performed 2-3 times a week at intervals of >48 hours. The participants were asked to keep posting in the WeChat group upon completion of the exercises. If the participant’s BG fluctuated greatly or had acute metabolic complications, the participant needed to wait until the condition was stabilized before gradually returning to exercise. We regularly evaluated whether the exercise program was suitable for the participant and made corresponding adjustments.

To reduce or avoid missed medications, the participants were also required to keep posting in the WeChat group after taking medications, and team members were responsible for the statistics. For participants who had not kept posting, they were notified by us on WeChat.

Wed-based health education was conducted between team members and participants. First, before the program was launched, our team members evaluated the participants’ knowledge of diabetes, medication use, insulin injection, BG monitoring, and diet and exercise. Second, we held T2DM self-management lectures regularly. Physicians, dietitians, diabetes specialist nurses, and trained diabetes health care educators in our team explained the T2DM-related knowledge, including pathogenesis; inducements; and the importance of regular BG monitoring, reasonable diet, medication administration, exercise, and correct BG monitoring methods. We reserved time during each lecture to encourage the participants to ask questions, and the team members answered patiently. After that, we held a T2DM health knowledge competition and encouraged the participants to interact with others. We gave rewards to the participants with outstanding performances. Finally, we conducted web-based health education through the WeChat group. We reminded the participants to take part in the interaction on time by sending messages and making phone calls, guided the participants to express their thoughts and experiences in the WeChat group, praised them for the right approach, and pointed out their mistakes. The focus was to guide the participants to realize the importance of regular medication, reasonable diet, proper exercise, and BG monitoring for glycemic control and to enhance the participants’ health awareness and self-management capabilities.

The participants were asked to record self-management data, including BG, meals, physical activity, and medication administration, and they sent this information to the team via WeChat. We reviewed the participants’ data daily, provided dietary recipes once a week, responded to participants’ and family members’ queries, and provided personalized feedback during each interaction. We also provided weekly and monthly summaries to the participants during follow-ups or through WeChat voice calls during the program.

### Outcome Measures

#### Overview

Outcome data were collected at baseline and 6 months after the intervention began. All participants were told to go to the community health care center for follow-up at the specified follow-up time and keep a fasting state. The primary outcome measure of the study was the change in fasting BG (FBG) and HbA_1c_ levels in the control and TangPlan and WeChat groups before and after the intervention. The main secondary outcomes included changes in body weight, BMI, BP, and serum lipid profiles. The above indicators were all determined by the laboratory department of the community health care center and by research assistants who did not know the grouping.

#### Anthropometric Parameters

When the participants arrived at the community health care center in a fasting state, their body weight and height were measured. Height was determined to the nearest 0.5 cm using a standard height gauge. BMI was calculated as weight (kg)/height (m)^2^.

#### Blood Test and BP

The nurses took venous blood samples for FBG, HbA_1c_, and serum lipid profile measurement. After distributing breakfast and instructing the participants to eat, blood was drawn again 2 hours later to measure 2-hour postload BG. During the waiting interval of the participants, their BP was measured after ensuring that the participants had been resting for at least 30 minutes.

### Statistical Analyses

Statistical analyses were performed using SPSS (version 22.0; IBM Corp). The Kolmogorov–Smirnov test or *Q*–*Q* plot was used to evaluate the normal distribution. Descriptive data are presented as mean and SD for continuous variables and frequency, with proportions for categorical variables. The 2-tailed independent sample *t* test and Mann–Whitney *U* test were used to assess the differences between groups of normally and nonnormally distributed data, respectively. The paired *t* test and Wilcoxon test were used to test the differences before and after the intervention for normally and nonnormally distributed data, respectively. According to the *Guidelines for the Prevention and Treatment of Type 2 Diabetes in China* proposed by the Chinese Diabetes Society in 2017, a reasonable HbA_1c_ control target is <7%. In treatment, HbA_1c_ ≥7% can be used as an important criterion for the initiation of clinical treatment of T2DM or the need to adjust the treatment plan. Accordingly, we divided the HbA_1c_ levels of the patients into two categories: HbA_1c_ <7% (normal) and HbA_1c_ ≥7% (abnormal). The McNemar test was used to determine the impact of the intervention on the HbA_1c_ levels of the control and TangPlan and WeChat groups before and after the intervention. *P*<.05 was considered statistically significant.

## Results

### Overview

**Figure 3:**
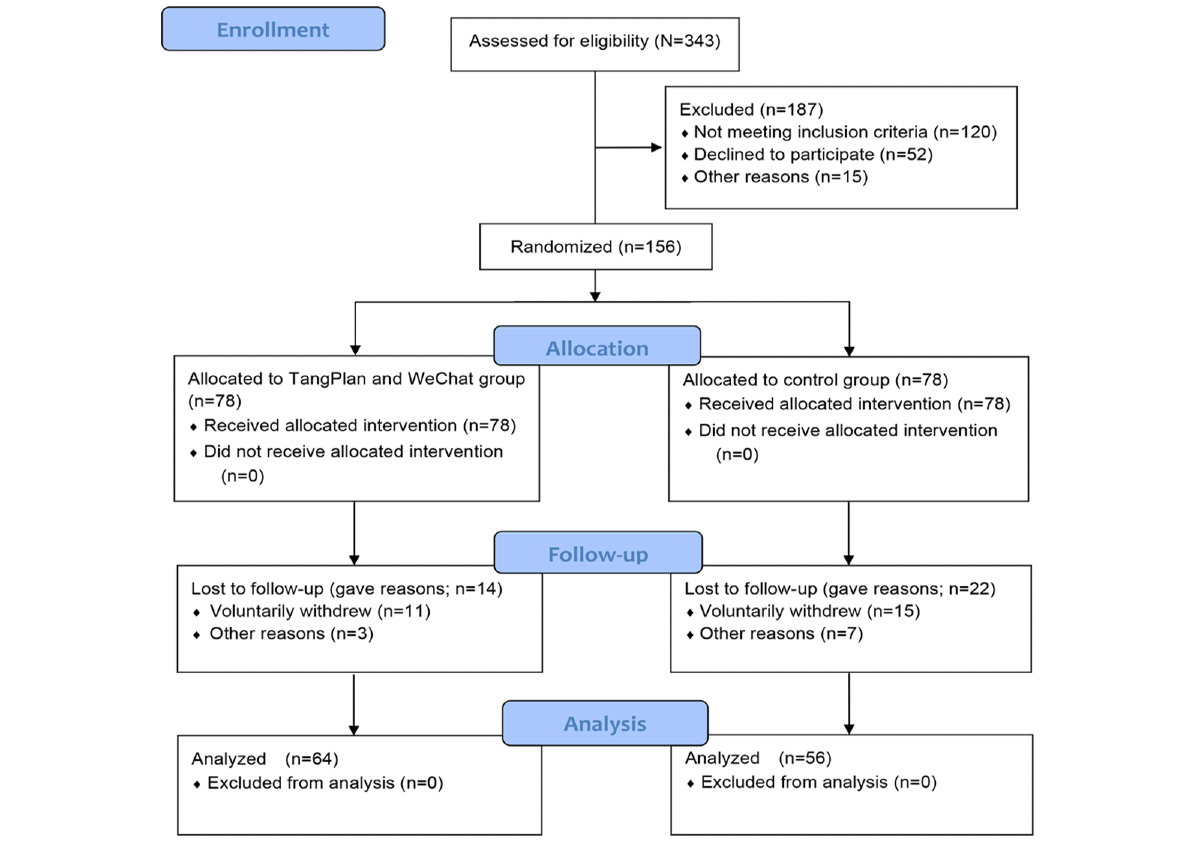
Participant flow diagram. HbA_1c_: glycated hemoglobin.

A total of 343 participants with T2DM were assessed for eligibility, of whom 187 (54.5%) were excluded (Figure 3). In total, 52 participants declined to take part because they were not interested in the program (18/52, 35%), did not want to pay much attention to diabetes (14/52, 27%), thought they did not need help (15/52, 29%), or had no reason (5/52, 10%). A total of 156 participants were randomized into the TangPlan and WeChat group (78/156, 50%) or the control group (78/156, 50%). Of these, 120 participants (TangPlan and WeChat: 64/120, 53.3%; control: 56/120, 46.7%) completed the follow-up assessments, yielding a retention rate of 76.9% (120/156).

### Baseline Characteristics of the Participants

The baseline characteristics of the participants are presented in Multimedia Appendix 3. Approximately 64% (36/56) male and 36% (20/56) female participants completed the trial in the control group, whereas 63% (40/64) male and 38% (24/64) female participants completed the trial in the TangPlan and WeChat group. No statistically significant differences in baseline characteristics, including age (*P*=.09), education level (*P*=.65), family monthly income (*P*=.49), T2DM duration in years (*P*=.66), body weight (*P*=.13), BMI (*P*=.10), HbA_1c_ (*P*=.84), FBG (*P*=.35), 2-hour postload BG (*P*=.36), serum lipid profiles, medication (*P*=.61), and presence of comorbidities (*P*=.76), were found between the control and TangPlan and WeChat groups.

### Changes in FBG and HbA_1c_

**Figure 4:**
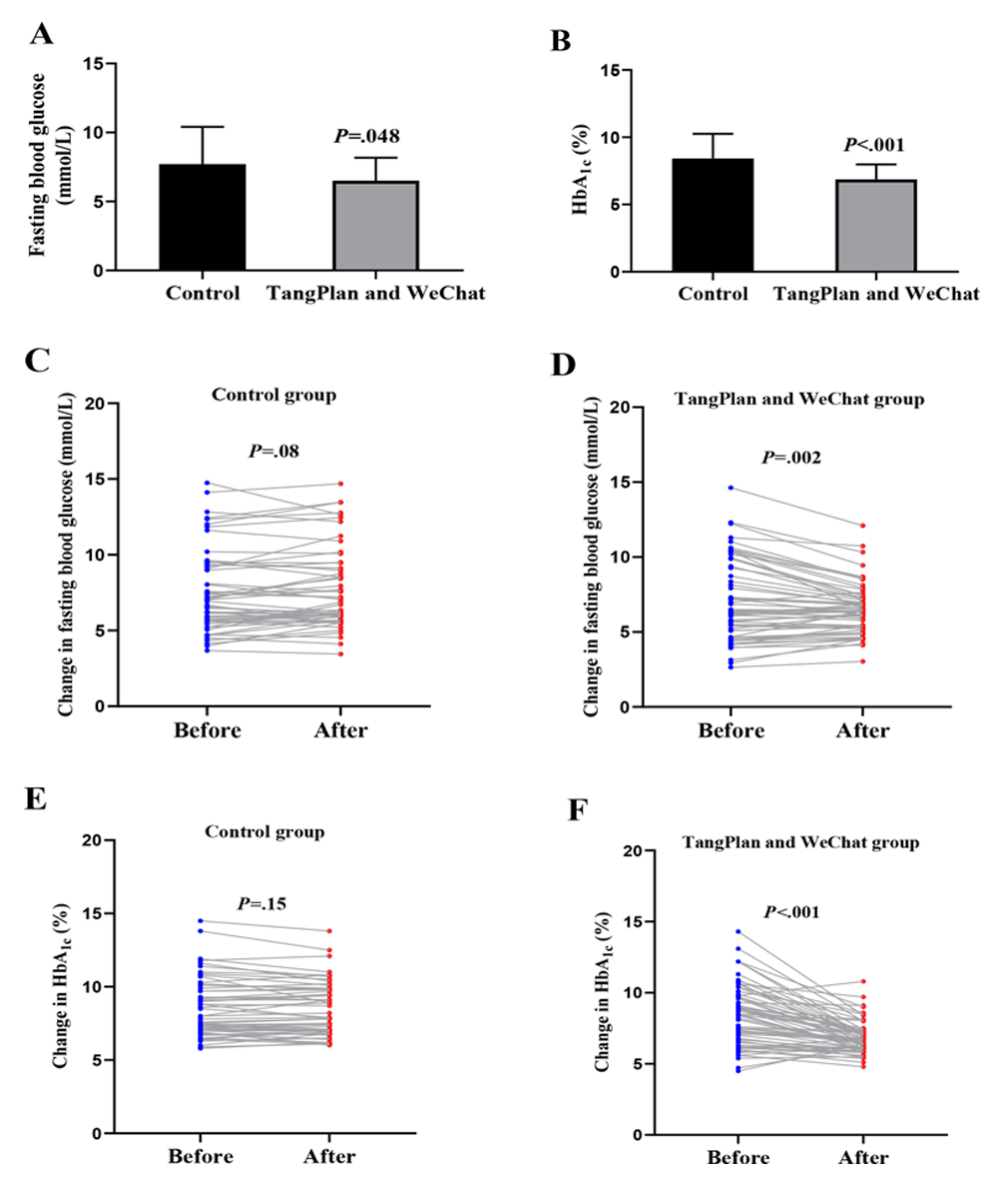
Comparison of fasting blood glucose and glycated hemoglobin (HbA_1c_) levels. (A) Fasting blood glucose difference between the control and TangPlan and WeChat groups after 6 months, (B) HbA_1c_ difference between the control and TangPlan and WeChat groups after 6 months, (C) fasting blood glucose changes in the control group at baseline and 6 months, (D) fasting blood glucose changes in the TangPlan and WeChat group at baseline and 6 months, (E) HbA_1c_ changes in the control group at baseline and 6 months, and (F) HbA_1c_ changes in the TangPlan and WeChat group at baseline and 6 months. In (A) and (B), data are shown as mean and SD.

After 6 months, the FBG (mean 6.51, SD 1.66 mmol/L) and HbA_1c_ (mean 6.87%, SD 1.11%) levels of the TangPlan and WeChat group were both significantly lower than those of the control group (FBG: mean 7.71, SD 2.70 mmol/L; HbA_1c_: mean 8.42%, SD 1.83%; Figure 4A and B). Compared with the baseline data, the mean change in FBG and HbA_1c_ in the control group was −0.21 mmol/L (SD 0.87 mmol/L; 95% CI −0.02 to 0.44 mmol/L; *P*=.08; Figure 4C) and −0.11% (SD 0.55%; 95% CI −0.26% to 0.04%; *P*=.15; Figure 4E), respectively, whereas the mean change in FBG and HbA_1c_ in the TangPlan and WeChat group was 0.51 mmol/L (SD 1.24 mmol/L; 95% CI −0.83 to −0.20 mmol/L; *P*=.002; Figure 4D) and −1.6% (SD 1.28%; 95% CI −1.92% to −1.28%; *P*<.001; Figure 4F), respectively.

In this trial, 23% (13/56) of participants had normal HbA_1c_ levels, and 77% (43/56) of participants had abnormal HbA_1c_ levels in the control group (Table 1). After 6 months of follow-up, there was no change in the number of abnormal and normal HbA_1c_ levels, but 15% (2/13) of the participants with normal HbA_1c_ levels demonstrated abnormal HbA_1c_ levels, and 5% (2/43) of the participants with abnormal HbA_1c_ levels had normal HbA_1c_ levels. The McNemar test revealed that there was no statistical difference in the proportion of participants with normal HbA_1c_ levels before and after the intervention (*P*=.99). In the TangPlan and WeChat group, 27% (17/64) of participants had normal HbA_1c_ levels, and 73% (47/64) of participants had abnormal HbA_1c_ levels before the intervention. After 6 months, 58% (37/64) of participants showed normal HbA_1c_ levels, and 42% (27/64) of participants had abnormal HbA_1c_ levels. Among them, 74% (20/27) of the participants with abnormal HbA_1c_ levels had normal HbA_1c_ levels. There was a significant difference in the proportion of participants with normal HbA_1c_ levels before and after the intervention (*P*<.001).

**Table 1:** Distribution of HbA_1c_ levels before and after the intervention (N=120).

Before the intervention		After the intervention, n (%)			*P* value
		HbA_1c_^a^<7%	HbA_1c_≥7%		
**Control group (n=56)**					.99
	HbA_1c_<7%	11 (19)	2 (4)		
	HbA_1c_ ≥7%	2 (4)	41 (73)		
**TangPlan and WeChat group (n=64)**					<.001
	HbA_1c_<7%	17 (27)	0 (0)		
	HbA_1c_ ≥7%	20 (31)	27 (42)		

^a^HbA_1c_: glycated hemoglobin.

### Changes in Body Weight, BMI, BP, and Serum Lipid Profiles

The body weight, BP, and serum lipid profiles of the participants at the end of the study are shown in Table 2. The body weight (mean 66.50, SD 9.51 kg) of the TangPlan and WeChat group was significantly lower than that of the control group (mean 71.91, SD 11.36 kg; *P*=.006), but no significant difference in BMI was observed (*P*=.10). Compared with the control group, the serum high-density lipoprotein cholesterol (HDL-C) level of the TangPlan and WeChat group (mean 1.20, SD 0.25 mmol/L) was significantly higher (*P*=.01), whereas systolic and diastolic BP (systolic BP: mean 127.03, SD 8.00 mm Hg and *P*=.005; diastolic BP: mean 75.25, SD 5.88 mm Hg and *P*=.03), serum low-density lipoprotein cholesterol (LDL-C; mean 2.50, SD 0.61 mmol/L; *P*=.006), and total cholesterol (TC; mean 4.01, SD 0.83 mmol/L; *P*=.02) levels in the TangPlan and WeChat group were significantly lower. In addition, the proportion of participants with HbA_1c_ <7% in the TangPlan and WeChat group was significantly higher than that in the control group (*P*<.001).

**Table 2:** Differences in body weight, BMI, blood pressure, and serum lipid profiles after 6 months (N=120).

Characteristic		Overall	Control group (n=56)	TangPlan and WeChat group (n=64)	*P* value	
Body weight (kg), mean (SD)		69.02 (10.72)	71.91 (11.36)	66.50 (9.51)	.006	
BMI (kg/m^2^), mean (SD)		23.67 (5.32)	24.53 (5.74)	22.92 (4.86)	.10	
SBP^a^ (mm Hg), mean (SD)		129.80 (10.22)	132.96 (11.56)	127.03 (8.00)	.005^b^	
DBP^c^ (mm Hg), mean (SD)		76.52 (6.87)	77.96 (7.64)	75.25 (5.88)	.03^b^	
**Serum lipid profiles (mmol/L), mean (SD)**						
	TG^d^	1.59 (0.83)	1.78 (1.10)	1.43 (0.40)	.33^b^	
	HDL-C^e^	1.15 (0.26)	1.08 (0.25)	1.20 (0.25)	.01	
	LDL-C^f^	2.71 (0.76)	2.96 (0.84)	2.50 (0.61)	.006^b^	
	TC^g^	4.22 (0.95)	4.42 (1.00)	4.01 (0.83)	.02	
**HbA_1c_^h^ distribution, n (%)**						<.001
	HbA_1c_ <7%	50 (41.7)	13 (23.2)	37 (57.8)		
	HbA_1c_ ≥7%	70 (58.3)	43 (76.8)	27 (42.2)		

^a^SBP: systolic blood pressure.

^b^Mann–Whitney *U* test was used.

^c^DBP: diastolic blood pressure.

^d^TG: triglycerides.

^e^HDL-C: high-density lipoprotein cholesterol.

^f^LDL-C: low-density lipoprotein cholesterol.

^g^TC: total cholesterol.

^h^HbA_1c_: glycated hemoglobin.

The mean changes in body weight, BP, and serum lipid profiles during the 6-month follow-up are illustrated in Table 3. Compared with the baseline data, the mean changes in body weight (*P*<.001), BMI (*P*<.001), systolic BP (*P*<.001), diastolic BP (*P*<.001), triglycerides (TG; *P*=.004), HDL-C (*P*=.001), LDL-C (*P*<.001), and TC (*P*<.001) in the TangPlan and WeChat group were significantly improved after the intervention, which was not observed in the control group.

**Table 3:** Changes in body weight, BMI, blood pressure, and serum lipid profiles during the 6-month follow-up (N=120).

Characteristic		Control group (n=56)		TangPlan and WeChat group (n=64)	
		Mean (SD; 95% CI)^a^	*P* value	Mean (SD; 95% CI)	*P* value
Body weight (kg)		−0.17 (0.91; −0.42 to 0.08)	.19^b^	−2.40 (2.84; −3.13 to −1.68)	<.001
BMI (kg/m^2^)		−0.06 (0.33; −0.15 to 0.03)	.09^b^	−0.85 (0.99; −1.10 to −0.60)	<.001^b^
SBP^c^ (mm Hg)		−0.16 (5.99; −1.76 to 1.44)	.84	−5.66 (6.87; −7.37 to −3.94)	<.001
DBP^d^ (mm Hg)		−0.66 (4.36; −1.83 to 0.51)	.26	−3.38 (4.57; −4.52 to −2.33)	<.001
**Serum lipid profiles (mmol/L)**					
	TG^e^	0.01 (0.11; −0.02 to 0.04)	.53	−0.09 (0.24; −0.16 to −0.03)	.004
	HDL-C^f^	−0.02 (0.95; −0.05 to 0.01)	.16	0.05 (0.10; 0.02 to 0.07)	.001
	LDL-C^g^	−0.01 (0.45; −0.13 to 0.11)	.88	−0.42 (0.46; −0.54 to −0.30)	<.001
	TC^h^	−0.03 (0.48; −0.16 to 0.11)	.68	−0.47 (0.51; −0.60 to −0.34)	<.001

^a^Mean was obtained by calculating the average of the values derived from the value after the intervention minus the value before the intervention for the same individual.

^b^Wilcoxon test was used.

^c^SBP: systolic blood pressure.

^d^DBP: diastolic blood pressure.

^e^TG: triglycerides.

^f^HDL-C: high-density lipoprotein cholesterol.

^g^LDL-C: low-density lipoprotein cholesterol.

^h^TC: total cholesterol.

## Discussion

### Principal Findings

This study assessed the effectiveness of the combination of the web-based TangPlan software and WeChat app in improving glycemic control (decrease in HbA_1c_ and BG levels) and serum lipid profiles (decline in TG, LDL-C, and TC and increase in HDL-C) as well as reducing body weight in patients with T2DM after 6 months of the program. Patients who used the TangPlan and WeChat–based intervention for 6 months reduced their HbA_1c_ levels by 1.6%, FBG levels by 0.51 mmol/L, body weight by 2.40 kg, BMI by 0.85 kg/m^2^, TG levels by 0.09 mmol/L, LDL-C levels by 0.42 mmol/L, and TC levels by 0.47 mmol/L and increased their HDL-C levels by 0.05 mmol/L.

Although many well-established methods of patient care have improved the clinical profile and complications associated with diabetes, the BG control rate is still at a low level. Owing to poor health education, the proportion of patients with diabetes who have optimal glycemic control (HbA_1c_ <7%) in China is <40%, and the rate is much lower in older adults [24,25]. With the inability to achieve control of BG, diabetes symptoms, and diabetes-related comorbidities through routine follow-up and patient self-management, interventions using mobile technology may improve the treatment effects of diabetes. As an adjuvant to standard self-management, some diabetes apps lead to a clinically remarkable reduction in HbA_1c_ levels [20], whereas some apps fail to improve HbA_1c_ levels [26]. However, although many diabetes apps have been developed in China, their use rate is only 15.44%. Use is higher among patients with type 1 diabetes than among patients with T2DM. The reasons why patients discontinue the use of an app include limited time (29.9%), complicated operations (25.4%), ineffectiveness for glycemic control (24.4%), and cost (19.3%) [27]. It has been reported that older patients have increased difficulty navigating and engaging with diabetes apps and are less likely to benefit from diabetes apps than younger patients [13].

WeChat is an extremely popular social app in China, and it is also easy to operate and can offer multiple functions, including texting and voice messages, free voice and video calls, group chats, and subscribing to public accounts. The value of WeChat in chronic disease management in China lies in that it can effectively overcome current difficulties such as time conflicts, geographic distance, and economic problems. This research in Henan Province, China, showed that offering health education through the WeChat platform for patients with diabetes by sending and explaining diabetes-related knowledge improved glycemic control [28]. Patients with diabetes who intervened through WeChat could receive a better education on BG self-monitoring, reasonable diet, exercise prescription, compliance with prescribed drugs, management of hypoglycemia and hyperglycemia, and weight control through communicating with nurses on WeChat. The frequency of communication between nurses and patients was 3 to 5 times in the first week, 2 to 4 times in the second week, and only once from the third week to the end. In the sixth month, the HbA_1c_ levels of the patients in the WeChat group were lower than those with usual care [29]. Another study with 2 years of follow-up demonstrated that WeChat could contribute to the establishment of a systematic health education model. Similar to what we did in this study, patients were also encouraged to monitor their BG, communicate regularly with the health education team via WeChat, and bring a record book to appointments, leading to lower HbA_1c_ levels and increasing compliance with the control criteria [30]. However, Chinese health care professionals are busy, and it is difficult for them to spend a lot of time giving patients with diabetes timely and complete self-management guidance only through WeChat. In this study, we designed a software named TangPlan for health care professionals, which contains multiple modules for diabetes management, especially the time-saving personalized nutrition customization module. TangPlan can help health care professionals take a short time to manage patients with diabetes, including automatically generating weekly personalized dietary recipes and recording BG monitoring values and exercise. In Chinese patients with poorly controlled diabetes, it was not easy to achieve long-term effective glucose improvement using app self-management alone, but combining it with wed-based management can help achieve rapid and sustained glycemic control [29]. The combination of WeChat and TangPlan in this trial brought benefits to patients with T2DM, which is suggested through improved HbA_1c_ levels, FBG levels, BP, and lipid profiles. However, we also need to enhance the interaction with patients to ensure timely tracking and feedback of the patients’ data.

The effectiveness of the intervention was evaluated through a significant reduction in HbA_1c_ levels. Studies have shown that a 0.5% to 1% reduction in HbA_1c_ levels is considered clinically significant and can reduce the risk of complications [31]. Even the Food and Drug Administration in the United States requires a 0.4% decline in HbA_1c_ levels for drug evaluation [32]. The results of the UK Prospective Diabetes Study showed that a 0.9% reduction in HbA_1c_ levels was related to a 25% decrease in microvascular complications, a 10% decrease in diabetes-related mortality, and a 6% reduction in all-cause mortality [33,34]. According to the evidence-based practice guidelines for diabetes in the United States, only 37% of persons with diabetes meet the HbA_1c_ target of <7%, and only 7% meet the combined glycemic, lipid, and BP goals [35]. We found that the combination of TangPlan and WeChat increased the proportion of patients with diabetes who met the HbA_1c_ target in the TangPlan and WeChat group from 27% (17/64) to 58% (37/64) after the intervention. This did not change in the control group. We believe that the average decline of 1.6% in HbA_1c_ levels in the TangPlan and WeChat group was significant in reducing the risk of diabetes-related complications and mortality.

FBG is another indicator of glycemic control and correlates with HbA_1c_ levels. A study showed that FBG levels >5.6 mmol/L but not of 3.9 to 5.6 mmol/L were associated with death [36]. Evidence indicates that a chronic hyperglycemic state is associated with impaired immunity [37], and FBG levels ≥7.0 mmol/L at admission are an independent predictor for 28-day mortality in patients with COVID-19 without a previous diagnosis of diabetes [38]. Hyperglycemia is also a risk factor for cardiovascular disease in T2DM [39]. The combination of TangPlan and WeChat remarkably decreased FBG levels, which indicated an additional benefit of reduction in cardiovascular risk among these patients.

Weight loss is one of the goals in the management of diabetes and is associated with improvements in HbA_1c_ levels [40]. The prospective Swedish Obese Subjects study revealed that weight reduction through gastric surgery performed on patients with obesity had a dramatic effect on the 8-year incidence of diabetes but no effect on the 8-year incidence of hypertension [41]. Each 5 kg/m^2^ decrease in BMI will prevent approximately 30% overall mortality in the population with diabetes [42]. The significant decrease in weight (2.40 kg) and BMI (0.85 kg/m^2^) among the TangPlan and WeChat group after 6 months of the program highlights the importance of weight reduction in ameliorating HbA_1c_ levels.

A variety of studies suggest that glucose alone is not responsible for diabetic complications, especially in individuals with T2DM [43,44]. Rather, the responsibility lies in a cluster of factors, including dyslipidemia, obesity, and hypertension, which have an impact on the adipose tissue fatty acid metabolism that underlines the onset and progression of diabetic complications [45]. The combination of TangPlan and WeChat exerted a beneficial effect on serum lipid profiles, illustrated by TG, TC, and LDL-C reduction and HDL-C elevation, further confirming the possibility of the TangPlan and WeChat intervention to reduce the incidence of diabetic complications.

To our knowledge, this study is one of the first to report the effectiveness of the combination of a self-designed diabetes management software and WeChat on glycemic control and other metabolic indicators. During the intervention process, whether it was during the follow-up, diabetes education lectures, or web-based communication via WeChat, there were frequent interactions between health care professionals and patients with diabetes, which might help promote a more harmonious relationship. However, one of the limitations of this trial was that the occurrence and progression of diabetes complications were not evaluated after the intervention. In addition, the program was performed for a short duration, and we did not independently quantify the influence of other behaviors and lifestyles on glycemic control. The loss of data during follow-up also limited the scope of this study. Future studies with a larger sample and better control will be able to further determine the effectiveness of the program.

### Conclusions

The combination of TangPlan and WeChat demonstrated an incremental decline in HbA_1c_ and FBG levels, body weight, and BP as well as improvements in serum lipid profiles during the 6-month program, indicating the feasibility, acceptance, and value of using a novel method for diabetes management. The results of this study can be further explored to assess the long-term acceptability, cost-effectiveness, and durability of the principal findings as well as the feasibility of the program in a larger population.

## Appendix

Multimedia Appendix 1 CONSORT-EHEALTH (Consolidated Standards of Reporting Trials of Electronic and Mobile Health Applications and Online Telehealth) checklist (V 1.6.1).

Multimedia Appendix 2 Weekly recipe of a participant (providing 1750 kcal).

Multimedia Appendix 3 Participant baseline characteristics at the start of the program.

## Abbreviations

BG: blood glucose
BP: blood pressure
CONSORT: Consolidated Standards of Reporting Trials
CONSORT-EHEALTH: Consolidated Standards of Reporting Trials of Electronic and Mobile Health Applications and Online Telehealth
DM: diabetes mellitus
FBG: fasting blood glucose
HbA_1c_: glycated hemoglobin
HDL-C: high-density lipoprotein cholesterol
LDL-C: low-density lipoprotein cholesterol
T2DM: type 2 diabetes mellitus
TC: total cholesterol
TG: triglycerides

## References

[1] (2017). IDF diabetes atlas. 8th edition. International Diabetes Federation.

[2] Wang L, Gao P, Zhang M, Huang Z, Zhang D, Deng Q, Li Y, Zhao Z, Qin X, Jin D, Zhou M, Tang X, Hu Y, Wang L (2017). Prevalence and ethnic pattern of diabetes and prediabetes in China in 2013. JAMA.

[3] National Commission of Health (2018). Chinese national statistical yearbook of health.

[4] Ryan EA, Holland J, Stroulia E, Bazelli B, Babwik SA, Li H, Senior P, Greiner R (2017). Improved A1C levels in type 1 diabetes with smartphone app use. Can J Diabetes.

[5] Devries JH, Snoek FJ, Heine RJ (2004). Persistent poor glycaemic control in adult type 1 diabetes. A closer look at the problem. Diabet Med.

[6] Blondon K, Klasnja P, Coleman K, Pratt W (2014). An exploration of attitudes toward the use of patient incentives to support diabetes self-management. Psychol Health.

[7] El-Gayar O, Timsina P, Nawar N, Eid W (2013). Mobile applications for diabetes self-management: status and potential. J Diabetes Sci Technol.

[8] Brzan PP, Rotman E, Pajnkihar M, Klanjsek P (2016). Mobile applications for control and self management of diabetes: a systematic review. J Med Syst.

[9] Garg SK, Shah VN, Akturk HK, Beatson C, Snell-Bergeon JK (2017). Role of mobile technology to improve diabetes care in adults with type 1 diabetes: the remote-T1D study iBGStar in type 1 diabetes management. Diabetes Ther.

[10] Hofer TP, Zemencuk JK, Hayward RA (2004). When there is too much to do: how practicing physicians prioritize among recommended interventions. J Gen Intern Med.

[11] Harris MI (2000). Health care and health status and outcomes for patients with type 2 diabetes. Diabetes Care.

[12] Given JE, O'Kane MJ, Bunting BP, Coates VE (2013). Comparing patient-generated blood glucose diary records with meter memory in diabetes: a systematic review. Diabet Med.

[13] Hou C, Carter B, Hewitt J, Francisa T, Mayor S (2016). Do mobile phone applications improve glycemic control (HbA1c) in the self-management of diabetes? A systematic review, meta-analysis, and GRADE of 14 randomized trials. Diabetes Care.

[14] Wu Y, Yao X, Vespasiani G, Nicolucci A, Dong Y, Kwong J, Li L, Sun X, Tian H, Li S (2018). Correction: mobile app-based interventions to support diabetes self-management: a systematic review of randomized controlled trials to identify functions associated with glycemic efficacy. JMIR Mhealth Uhealth.

[15] Kebede MM, Pischke CR (2019). Corrigendum: popular diabetes apps and the impact of diabetes app use on self-care behaviour: a survey among the digital community of persons with diabetes on social media. Front Endocrinol (Lausanne).

[16] Chan JC, Zhang Y, Ning G (2014). Diabetes in China: a societal solution for a personal challenge. Lancet Diabetes Endocrinol.

[17] Trawley S, Baptista S, Browne JL, Pouwer F, Speight J (2017). The use of mobile applications among adults with type 1 and type 2 diabetes: results from the second MILES-Australia (MILES-2) study. Diabetes Technol Ther.

[18] Chen X, Zhou X, Li H, Li J, Jiang H (2020). The value of WeChat application in chronic diseases management in China. Comput Methods Programs Biomed.

[19] He C, Wu S, Zhao Y, Li Z, Zhang Y, Le J, Wang L, Wan S, Li C, Li Y, Sun X (2017). Social media-promoted weight loss among an occupational population: cohort study using a WeChat mobile phone app-based campaign. J Med Internet Res.

[20] Shi B, Liu X, Dong Q, Yang Y, Cai Z, Wang H, Yin D, Wang H, Dou K, Song W (2021). The effect of a WeChat-based tertiary A-level hospital intervention on medication adherence and risk factor control in patients with stable coronary artery disease: multicenter prospective study. JMIR Mhealth Uhealth.

[21] Jiang Y, Liu F, Guo J, Sun P, Chen Z, Li J, Cai L, Zhao H, Gao P, Ding Z, Wu X (2020). Evaluating an intervention program using WeChat for patients with chronic obstructive pulmonary disease: randomized controlled trial. J Med Internet Res.

[22] Eysenbach G, CONSORT-EHEALTH Group (2011). CONSORT-EHEALTH: improving and standardizing evaluation reports of web-based and mobile health interventions. J Med Internet Res.

[23] Güner TA, Coşansu G (2020). The effect of diabetes education and short message service reminders on metabolic control and disease management in patients with type 2 diabetes mellitus. Prim Care Diabetes.

[24] Chen R, Ji L, Chen L, Chen L, Cai D, Feng B, Kuang H, Li H, Li Y, Liu J, Shan Z, Sun Z, Tian H, Xu Z, Xu Y, Yang Y, Yang L, Yu X, Zhu D, Zou D (2015). Glycemic control rate of T2DM outpatients in China: a multi-center survey. Med Sci Monit.

[25] Ji LN, Lu JM, Guo XH, Yang WY, Weng JP, Jia WP, Zou DJ, Zhou ZG, Yu DM, Liu J, Shan ZY, Yang YZ, Hu RM, Zhu DL, Yang LY, Chen L, Zhao ZG, Li QF, Tian HM, Ji QH, Liu J, Ge JP, Shi LX, Xu YC (2013). Glycemic control among patients in China with type 2 diabetes mellitus receiving oral drugs or injectables. BMC Public Health.

[26] Castensøe-Seidenfaden P, Husted GR, Jensen AK, Hommel E, Olsen B, Pedersen-Bjergaard U, Kensing F, Teilmann G (2018). Testing a smartphone app (Young with Diabetes) to improve self-management of diabetes over 12 months: randomized controlled trial. JMIR Mhealth Uhealth.

[27] Zhang Y, Li X, Luo S, Liu C, Xie Y, Guo J, Liu F, Zhou Z (2019). Use, perspectives, and attitudes regarding diabetes management mobile apps among diabetes patients and diabetologists in China: national web-based survey. JMIR Mhealth Uhealth.

[28] Zhang Y, Chu L (2018). Effectiveness of systematic health education model for type 2 diabetes patients. Int J Endocrinol.

[29] Zhang L, He X, Shen Y, Yu H, Pan J, Zhu W, Zhou J, Bao Y (2019). Effectiveness of smartphone app-based interactive management on glycemic control in Chinese patients with poorly controlled diabetes: randomized controlled trial. J Med Internet Res.

[30] Dong Y, Wang P, Dai Z, Liu K, Jin Y, Li A, Wang S, Zheng J (2018). Increased self-care activities and glycemic control rate in relation to health education via WeChat among diabetes patients: a randomized clinical trial. Medicine (Baltimore).

[31] Nathan DM, Buse JB, Davidson MB, Heine RJ, Holman RR, Sherwin R, Zinman B, Professional Practice Committee‚ American Diabetes Association, European Association for the Study of Diabetes (2006). Management of hyperglycaemia in type 2 diabetes: a consensus algorithm for the initiation and adjustment of therapy. A consensus statement from the American Diabetes Association and the European Association for the Study of Diabetes. Diabetologia.

[32] American Diabetes Association (2020). 6. Glycemic targets: standards of medical care in diabetes-2020. Diabetes Care.

[33] Chrvala CA, Sherr D, Lipman RD (2016). Diabetes self-management education for adults with type 2 diabetes mellitus: a systematic review of the effect on glycemic control. Patient Educ Couns.

[34] Katulanda P, Jayawardena R, Jayawardana R, Ranasinghe P, Rezvi Sheriff MH, Matthews DR (2013). Physical activity patterns and correlates among adults from a developing country: the Sri Lanka Diabetes and Cardiovascular Study. Public Health Nutr.

[35] American Diabetes Association (2008). Economic costs of diabetes in the U.S. In 2007. Diabetes Care.

[36] Rao Kondapally Seshasai S, Kaptoge S, Thompson A, Di Angelantonio E, Gao P, Sarwar N, Whincup PH, Mukamal KJ, Gillum RF, Holme I, Njølstad I, Fletcher A, Nilsson P, Lewington S, Collins R, Gudnason V, Thompson SG, Sattar N, Selvin E, Hu FB, Danesh J, Emerging Risk Factors Collaboration (2011). Diabetes mellitus, fasting glucose, and risk of cause-specific death. N Engl J Med.

[37] Pearson-Stuttard J, Blundell S, Harris T, Cook DG, Critchley J (2016). Diabetes and infection: assessing the association with glycaemic control in population-based studies. Lancet Diabetes Endocrinol.

[38] Wang S, Ma P, Zhang S, Song S, Wang Z, Ma Y, Xu J, Wu F, Duan L, Yin Z, Luo H, Xiong N, Xu M, Zeng T, Jin Y (2020). Fasting blood glucose at admission is an independent predictor for 28-day mortality in patients with COVID-19 without previous diagnosis of diabetes: a multi-centre retrospective study. Diabetologia.

[39] Haffner SJ, Cassells H (2003). Hyperglycemia as a cardiovascular risk factor. Am J Med.

[40] Gummesson A, Nyman E, Knutsson M, Karpefors M (2017). Effect of weight reduction on glycated haemoglobin in weight loss trials in patients with type 2 diabetes. Diabetes Obes Metab.

[41] Sjöström CD, Peltonen M, Wedel H, Sjöström L (2000). Differentiated long-term effects of intentional weight loss on diabetes and hypertension. Hypertension.

[42] Cheng Y, Zhang H, Chen R, Yang F, Li W, Chen L, Lin S, Liang G, Cai D, Chen H (2014). Cardiometabolic risk profiles associated with chronic complications in overweight and obese type 2 diabetes patients in South China. PLoS One.

[43] Duckworth W, Abraira C, Moritz T, Reda D, Emanuele N, Reaven PD, Zieve FJ, Marks J, Davis SN, Hayward R, Warren SR, Goldman S, McCarren M, Vitek ME, Henderson WG, Huang GD, VADT Investigators (2009). Glucose control and vascular complications in veterans with type 2 diabetes. N Engl J Med.

[44] Zoungas S, Arima H, Gerstein HC, Holman RR, Woodward M, Reaven P, Hayward RA, Craven T, Coleman RL, Chalmers J, Collaborators on Trials of Lowering Glucose (CONTROL) group (2017). Effects of intensive glucose control on microvascular outcomes in patients with type 2 diabetes: a meta-analysis of individual participant data from randomised controlled trials. Lancet Diabetes Endocrinol.

[45] Callaghan BC, Xia R, Banerjee M, de Rekeneire N, Harris TB, Newman AB, Satterfield S, Schwartz AV, Vinik AI, Feldman EL, Strotmeyer ES, Health ABC Study (2016). Metabolic syndrome components are associated with symptomatic polyneuropathy independent of glycemic status. Diabetes Care.

