# Supplementary figures and images for: Web-Based TangPlan and WeChat Combination to Support Self-management for Patients With Type 2 Diabetes: Randomized Controlled Trial
Source: JMIR Mhealth Uhealth. 2022 Mar 30;10(3):e30571. doi: 10.2196/30571 (PMC9008529; doi:10.2196/30571)

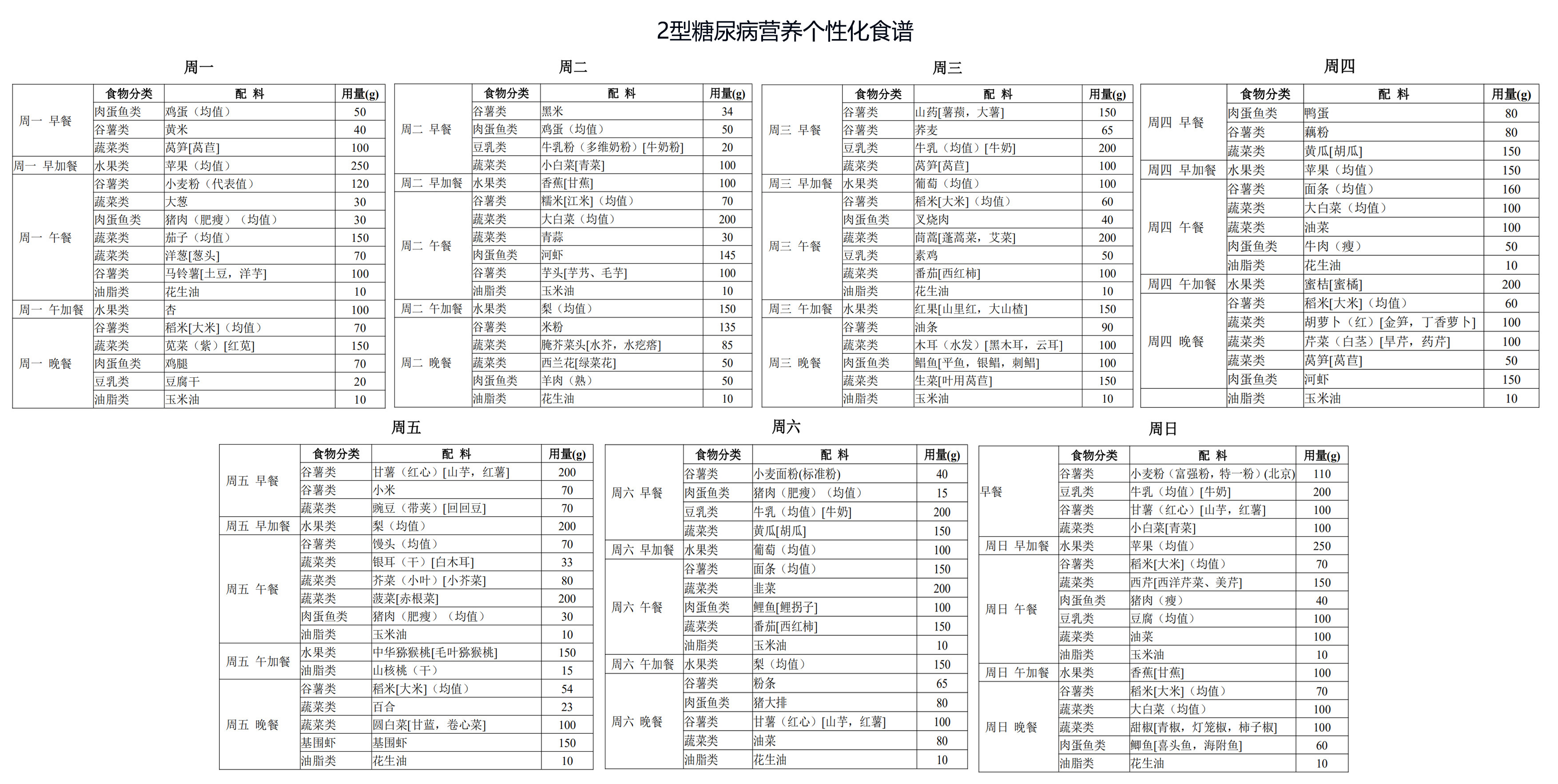

Supplement: Multimedia Appendix 2 [file mhealth_v10i3e30571_app2.png]
