# Supplementary material for: Web-Based TangPlan and WeChat Combination to Support Self-management for Patients With Type 2 Diabetes: Randomized Controlled Trial
Source: JMIR Mhealth Uhealth. 2022 Mar 30;10(3):e30571. doi: 10.2196/30571 (PMC9008529; doi:10.2196/30571)
Supplement: Multimedia Appendix 3 [file mhealth_v10i3e30571_app3.docx]

Multimedia Appendix 3. Participant baseline characteristics at the start of the program.

|  | Overall  (n = 120) | Control group  (n = 56) | TWC group  (n = 64) | *t* / χ^2^ | *P* |
| --- | --- | --- | --- | --- | --- |
| **Gender, n (%)** |  |  |  |  |  |
| Male | 76 (63) | 36 (64) | 40 (63) | 0.04 | .84 |
| Female | 44 (37) | 20 (36) | 24 (37) |  |  |
| Age (years), mean (SD) | 60.04 (12.56) | 57.96 (12.30) | 61.86 (12.59) | 1.71 | .09 |
| **Education level, n (%)** |  |  |  |  |  |
| Primary school and below | 28 (23) | 11 (20) | 17 (27) | 1.64 | .65 |
| Middle school | 44 (37) | 21 (37) | 23 (36) |  |  |
| High school or secondary school | 29 (24) | 16 (29) | 13 (20) |  |  |
| Junior college or above | 19 (16) | 8 (14) | 11 (17) |  |  |
| **Family monthly income (RMB), n (%)** |  |  |  |  |  |
| <2000 | 12 (10) | 4 (7) | 8 (12) | 1.44 | .49 |
| 2000~5000 | 40 (33) | 21 (38) | 19 (30) |  |  |
| >5000 | 68 (57) | 31 (55) | 37 (58) |  |  |
| T2DM duration (years), mean (SD) | 10.44 (8.07) | 10.09 (7.09) | 10.75 (8.88) | 0.44 | .66 |
| Body weight (kg), mean (SD) | 70.38 (11.32) | 72.07 (11.14) | 68.90 (11.36) | -1.51 | .13 |
| BMI^a^ (kg/m^2^), mean (SD) | 24.97 (3.24) | 25.50 (3.17) | 24.51 (3.26) | -1.64 | .10 |
| HbA_1c_^b^ (%), mean (SD) | 8.54 (2.07) | 8.58 (2.02) | 8.50 (2.13) | -0.20 | .84 |
| **HbA_1c_ distribution, n (%)** |  |  |  |  |  |
| HbA_1c_ < 7% | 30 (25) | 13 (23) | 17 (27) | 0.18 | .67 |
| HbA1c ≥ 7% | 90 (75) | 43 (77) | 47 (73) |  |  |
| SBP^c^ (mmHg), mean (SD) | 132.89 (12.64) | 133.13 (14.09) | 132.69 (13.34) | -0.18 | .86 |
| DBP^d^ (mmHg), mean (SD) | 78.63 (9.50) | 78.63 (9.97) | 78.63 (9.14) | 0.00 | 1.00 |
| FBG^e^ (mmol/L), mean (SD) | 7.25 (2.69) | 7.50 (2.74) | 7.03 (2.64) | -0.94 | .35 |
| 2hBG^f^ (mmol/L), mean (SD) | 12.90 (4.35) | 12.47 (4.16) | 13.25 (4.50) | 0.99 | .36 |
| **Serum lipid profiles (mmol/L), mean (SD)** | | | | | |
| TG^g^ | 1.64 (0.90) | 1.77 (1.15) | 1.52 (0.58) | -1.46 | .15 |
| HDL-C ^h^ | 1.13 (0.30) | 1.10 (0.29) | 1.16 (0.30) | 0.98 | .33 |
| LDL-C^i^ | 2.94 (0.91) | 2.97 (0.91) | 2.92 (0.92) | -0.28 | .78 |
| TC^j^ | 4.49 (1.18) | 4.44 (1.15) | 4.52 (1.20) | 0.34 | .73 |
| **Medication, n (%)** |  |  |  |  |  |
| Oral hypoglycemic agents | 44 (37) | 22 (39) | 22 (34) | 0.99 | .61 |
| Insulin | 21 (17) | 11 (20) | 10 (16) |  |  |
| Both | 55 (46) | 23 (41) | 32 (50) |  |  |
| **Presence of comorbidities, n (%)** |  |  |  |  |  |
| Yes | 81 (68) | 37 (66) | 44 (69) | 0.10 | .76 |
| No | 39 (32) | 19 (34) | 20 (31) |  |  |

^a^BMI: body mass index.

^b^HbA_1c_: glycated hemoglobin.

^c^SBP: systolic blood pressure.

^d^DBP: diastolic blood pressure.

^e^FBG: fasting blood glucose.

^f^2hBG: 2-h blood glucose.

^g^TG: triglycerides.

^h^HDL-C: high-density lipoprotein cholesterol.

^i^LDL-C: low-density lipoprotein cholesterol.

^j^TC: total cholesterol.
